# Using a human cardiovascular-respiratory model to characterize cardiac tamponade and pulsus paradoxus

**DOI:** 10.1186/1742-4682-6-15

**Published:** 2009-08-06

**Authors:** Deepa Ramachandran, Chuan Luo, Tony S Ma, John W Clark

**Affiliations:** 1 Department of Electrical and Computer Engineering, Rice University, Houston, Texas 77005, USA; 2 Division of Cardiology, VA Medical Center, Houston, Texas 77030, USA; 3 Baylor College of Medicine, One Baylor Plaza, Houston, Texas 77030, USA

## Abstract

**Background:**

Cardiac tamponade is a condition whereby fluid accumulation in the pericardial sac surrounding the heart causes elevation and equilibration of pericardial and cardiac chamber pressures, reduced cardiac output, changes in hemodynamics, partial chamber collapse, pulsus paradoxus, and arterio-venous acid-base disparity. Our large-scale model of the human cardiovascular-respiratory system (H-CRS) is employed to study mechanisms underlying cardiac tamponade and pulsus paradoxus. The model integrates hemodynamics, whole-body gas exchange, and autonomic nervous system control to simulate pressure, volume, and blood flow.

**Methods:**

We integrate a new pericardial model into our previously developed H-CRS model based on a fit to patient pressure data. Virtual experiments are designed to simulate pericardial effusion and study mechanisms of pulsus paradoxus, focusing particularly on the role of the interventricular septum. Model differential equations programmed in C are solved using a 5^th^-order Runge-Kutta numerical integration scheme. MATLAB is employed for waveform analysis.

**Results:**

The H-CRS model simulates hemodynamic and respiratory changes associated with tamponade clinically. Our model predicts effects of effusion-generated pericardial constraint on chamber and septal mechanics, such as altered right atrial filling, delayed leftward septal motion, and prolonged left ventricular pre-ejection period, causing atrioventricular interaction and ventricular desynchronization. We demonstrate pericardial constraint to markedly accentuate normal ventricular interactions associated with respiratory effort, which we show to be the distinct mechanisms of pulsus paradoxus, namely, series and parallel ventricular interaction. Series ventricular interaction represents respiratory variation in right ventricular stroke volume carried over to the left ventricle via the pulmonary vasculature, whereas parallel interaction (via the septum and pericardium) is a result of competition for fixed filling space. We find that simulating active septal contraction is important in modeling ventricular interaction. The model predicts increased arterio-venous CO_2 _due to hypoperfusion, and we explore implications of respiratory pattern in tamponade.

**Conclusion:**

Our modeling study of cardiac tamponade dissects the roles played by septal motion, atrioventricular and right-left ventricular interactions, pulmonary blood pooling, and the depth of respiration. The study fully describes the physiological basis of pulsus paradoxus. Our detailed analysis provides biophysically-based insights helpful for future experimental and clinical study of cardiac tamponade and related pericardial diseases.

## Background

Cardiac tamponade is a condition whereby the accumulation of fluid in the pericardial sac causes a hemodynamically significant in the intra-pericardial pressure (P_PERI_) which is conventionally defined as a liquid pressure. In a healthy subject, P_PERI _is approximately equal to the pleural pressure (P_PL_). P_PERI _rises with increasing effusion and may equalize to diastolic right atrial (RA) and right ventricular (RV) pressures, and at higher levels of effusion to diastolic left atrial (LA) and left ventricular (LV) pressures. Heightened pericardial pressure may lead to partial chamber collapse for a portion of the cardiac cycle [1,2] wherein P_PERI _exceeds chamber pressure. Clinical cardiac tamponade occurs when there is significant component of decreased cardiac output, stroke volume, systemic blood pressure, attendant tachycardia, and manifestation of pulsus paradoxus (an exaggerated respiratory fluctuation of systolic pressure by a greater amount than 10 mmHg or 10% [3]).

Cardiac tamponade may present as an acute clinical emergency or in a less emergent fashion that requires timely intervention [4]. Low-pressure tamponade has also been described [5]. Here we demonstrate a case of virtual subacute tamponade, modeled on the hemodynamic data reported by Reddy et al. [3] concerning a case of tamponade requiring pericardiocentesis.

Pericardial effusion leads to increased chamber interaction. A *parallel *interaction occurs whereby expansion of the RV during inspiration compresses the LV; likewise, a smaller RV volume during expiration allows more blood to be drawn into the LV [6-10]. The septum and pericardium are involved in this interaction. The septum is driven directionally by the prevailing pressure gradient across it, but is not a passive interventricular partition; it acts as a contractile pump in its own right [11-14]. Localized chamber pressure changes are transferred throughout the heart via the surrounding effusion-filled pericardium [7,15] aiding chamber interaction. An exaggerated *series *form of ventricular interaction occurs in tamponade when an augmented right heart volume upon inspiration travels to the left heart within two to three beats, contributing to an increase in LV stroke volume (LVSV) at the expiratory phase of respiration [16,17]. Parallel and series ventricular interaction have been hypothesized to be the important mechanisms involved in the generation of pulsus paradoxus [3,9,16-18] but their individual contributions have not been quantified. Additionally, atrioventricular (AV) interaction [19] causes systole-dominant atrial filling in the setting of elevated pericardial constraint and may change the filling patterns of all four chambers. We show that in severe tamponade this mechanism can lead to lowered filling volumes that changes septal motion and affects ventricular ejection times. AV interaction thus plays an important role in the generation of pulsus paradoxus.

### Human Cardiovascular Respiratory System (H-CRS) Model

Large-scale integrated cardiovascular-respiratory closed-loop models provide informative analysis of normal and diseased human physiology [11,12,20-27], since they can capture the global aspects of cardiovascular-respiratory interactions. Our group has developed a model of the human cardiovascular respiratory system (H-CRS) that integrates hemodynamics, whole-body and cerebral gas exchange, and baro- and chemoreceptor reflexes. This model accurately simulates the complex ventricular and cardio-respiratory interactions that occur during the Valsalva maneuver [24], apnea [25], left ventricular diastolic dysfunction [11], and interventricular septal motion [12]. Here, we update our composite model of the human subject with an appropriate pericardial pressure-volume characteristic to better simulate chronic cardiac tamponade.

Sun et al. [27] have modeled tamponade in a closed-loop, baroreflex-controlled, circulatory model by incorporating right-left heart interaction via a septal elastic compartment. Their septum is limited to a passive coupling of the ventricles via the ventricular pressure gradient. With a completely passive septum, septal motion could not oppose the established trans-septal pressure gradient. Our H-CRS model contains a septal subsystem model that is both active and passive in that it acts as a contractile pump that assists left chamber ejection and the RV in filling. We hold this to be a key distinction, in that biphasic septal motion has been demonstrated experimentally in normal hearts [13,14] and our simulations show that in tamponade it can be an important contributing factor to systolic operation. Additionally, their pulmonary component does not model pulmonary mechanics or pulmonary circulatory changes as a function of breathing movements, but is limited to a specification of pleural pressure drive. These circulatory changes mediated by respiration are important in tamponade and especially in the production of pulsus paradoxus, as will be shown. Finally, our model demonstrates important physiological alterations of gas exchange in the setting of cardiac tamponade.

In this work, we first examine the model-generated predictions of cardiovascular pressures, volumes, and flows in tamponade, with particular focus on the role of an active septum. We then analyze the contributory role of breathing pattern, and by introducing artificial isolation of the right and left hearts, dissect the separate contributions of serial and parallel ventricular interactions. Lastly, we analyze the important role of the septum as an active, tertiary pump assisting both systolic ejection and diastolic filling, and demonstrate the relevance of this previously neglected component in the physiology of cardiac tamponade.

## Methods

### H-CRS Model

Our H-CRS model [11,12,24-26] has three major parts: models of the cardiovascular, respiratory, and neural control systems. The cardiovascular component includes a lumped pump-type model of the heart chambers, lumped models of the inlet and outlet valves, as well as the systemic and pulmonary circulations considered as pump loads. Specifically, the walls of the heart chambers and septum are described in terms of time-varying elastance functions. The pericardium enveloping the heart is modeled as a passive nonlinear elastic membrane enclosing the pericardial fluid volume. Distributed models of the systemic, pulmonary, and cerebral circulations are included as previously described [11] and nonlinear pressure-volume (P-V) relationships are used to describe the peripheral venous system. The respiratory element in the H-CRS model includes lumped models of lung mechanics and gas transport, which are coupled with the pulmonary circulation model. Specifically, the nonlinear resistive-compliant properties of the airways are described as well as the nonlinear P-V relationship of the lungs. In the pulmonary circulation model, pulmonary capillary transmural pressure (hence volume) is dependent on alveolar pressure, whereas pulmonary arterial and venous transmural pressures are dependent on pleural pressure [28]. Whole-body gas transport is included in the respiratory element with gas exchange equations given for each gaseous species (O_2_, CO_2_, and N_2_) at the lung and in major tissues of the body at the capillary level (i.e., skeletal muscle and brain). The neural control system model includes baroreceptor control of heart rate, contractility, and vasomotor tone, and chemoreceptor control of heart rate and vasomotor tone [24]. Parameters associated with the systemic and pulmonary circulations have been adjusted to fit typical input impedance data (systemic and pulmonary) from normal human patients [11].

Differential equations for the H-CRS model were programmed in C and solved numerically using a 5^th^-order Cash-Karp Runge-Kutta method [29]. Typically, a 50-second simulation required a run time of five minutes on an AMD Turion 1.6-GHz platform (Dell Inspiron 1501). Specific modifications made to the H-CRS model for this study of tamponade and pulsus paradoxus are described in the subsections below.

#### Pericardial Model

**Figure 1:**
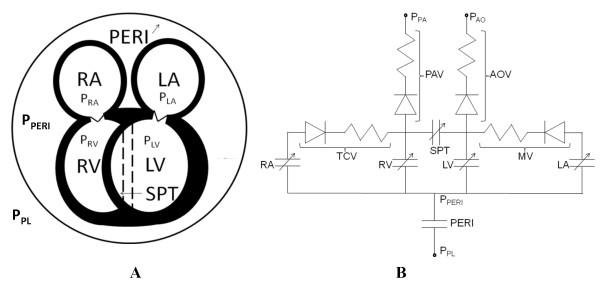
**Five-Compartment Heart Model**. Panel A shows the five-compartment heart model. An elastic pericardium encloses all four heart chambers. The dotted lines represent septal position when relaxed. Panel B is the equivalent hydraulic circuit model. Anatomical components of the equivalent circuit (LV = left ventricle, RV = right ventricle, LA = left atrium, RA = right atrium, SPT = interventricular septum, PERI = pericardium, TCV = tricuspid valve, MV = mitral valve, AOV = aortic valve, PAV = pulmonary valve). Specific pressures (P_PL _= pleural pressure, P_PA _= pulmonary arterial pressure, P_AO _= aortic pressure, P_PERI _= pericardial pressure, P_RA _= right atrial pressure, P_LA _= left atrial pressure, P_RV _= right ventricular pressure, P_LV _= left ventricular pressure).

The H-CRS model [11] is updated with a modified pericardial element. Figure 1A shows our five-compartment heart model, with the four chambers enclosed by the pericardium and a separate septum. Figure 1B is a hydraulic equivalent circuit of the heart model. The modification consists in specification of a transmural pericardial pressure (P_TPERI_) vs. pericardial effusion volume (V_PERI_) relationship, where P_TPERI _is defined as P_PERI _minus P_PL_. A nonlinear least-squares parameter estimation method [30] was used to obtain the the transmural pericardial pressure – to – pericardial volume relationship by adopting the P_PERI _vs. V_PERI _data from a clinical case reported by Reddy et al. [3]. Effusion levels up to 600 ml were assumed to have no effect on the pericardium in chronic tamponade, and a normal pressure-volume response was modeled for this range. P_TPERI _was calculated from this data under the assumption of a constant mean P_PL _of -3.0 mmHg. This new P_TPERI_-V_PERI _relationship is given by Eq. 1, where P_0 _(= 4.24e-7 mmHg) is the P_PERI _coefficient, λ (= 0.0146 ml^-1^) the pericardial stiffness parameter, V_PERI _the effusion volume, V_H _the total heart volume, and V_0 _(=159.36 ml) the volume offset:

(1)

The new and old transmural pressure-volume characteristics of the pericardial space differ in that their slopes in the normal range of volumes are approximately the same, however at high volumes, the new characteristic develops significantly greater pressures.

#### Respiratory Model

Apart from gas exchange modeled in the lung and airways [11], time-varying pleural pressure due to breathing is also simulated in the respiratory section of the model. In order to better characterize the cardio-respiratory interactions in tamponade, we employed a spontaneous tidal breathing waveform digitized from a canine study of tamponade [17] and scaled it to human proportions of mean P_PL _-3.0 mmHg. This pseudo-human respiratory waveform has P_PL _range estimated from [3] and [31].

#### Septal Model

Three septal models were compared: two passive septa, whose P-V relationship was fixed at either end-systolic or end-diastolic behavior throughout the cardiac cycle, and an active septum for which the P-V relationship is modulated by a time-dependent activation function in synchrony with free wall contraction, thereby undergoing biphasic operation. The passive septum models are used only for this comparison study – all simulations of control and tamponade employ the active septum model detailed previously [11,12].

### Virtual Experiments

#### Cardiac Tamponade

Tamponade was simulated by graded increases in pericardial volume. Following each step-increase, the model was brought to steady-state and data was analyzed using MATLAB [30]. Effusion levels from 15 ml to 1100 ml were used. We consider effusion of 15 ml as control case, 900 ml as moderate tamponade, and 1000 ml as severe tamponade.

#### Pulsus Paradoxus: Ventricular Interaction Studies

To analyze ventricular interaction, we tracked a fixed volume of blood as it was transported from the right atrium to left ventricle. In Experiment 1 (see Results section), we simulated an inspiratory increase in venous return to the right heart by delivering a triangular pulse volume to the vena cava within a period of two seconds at fixed P_PL_.

In Experiment 2, to dissect the relative importance of each type of ventricular interaction, the model was modified to eliminate one type of interaction at a time (see Experiment 2 in Results section). To study series interaction, parallel interactions via the septum and pericardium were respectively eliminated by increasing the septal stiffness parameter λ by 100× from 0.05 to 5.0, and holding P_PERI _constant. To study parallel interaction, the pulmonary venous volume was held constant thus creating an independent left heart venous return, thereby eliminating series interaction. Parallel and series ventricular interactions were analyzed and compared based on a triangular pulse of venous return to the right atrium such as in Experiment 1, and P_PL _was held constant.

## Results

### Effects of Pericardial Effusion

#### Equilibration of Diastolic Pressures and Chamber Collapse

**Figure 2:**
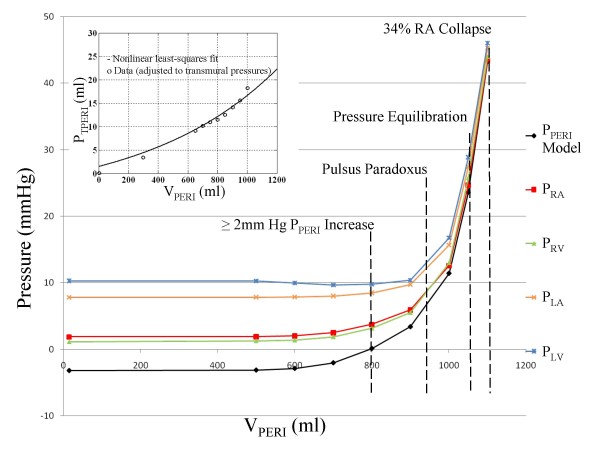
**Pressure-Volume Relationship**. Various pressures as a function of pericardial effusion volume V_PERI_. These pressures include pericardial pressure (P_PERI_), mean diastolic atrial (P_RA _and P_LA_) and ventricular (P_RV _and P_LV_) pressures. At 800 ml, there is a 2 mmHg increase in pericardial pressure and equalization to right diastolic chamber pressures. At 950 ml, pulsus paradoxus first appears. At 1050 ml, chamber pressures equalize to within 2 mmHg of each other and chamber collapse is observed at 1100 ml, with 34% of the mean cardiac cycle marked by collapse of the right atrium. The figure insert plot (top left) shows the transmural pericardial pressure vs. pericardial volume for data points derived from Reddy et al. [3] in which a fixed mean pleural pressure is assumed, and a nonlinear least-squares fit to the data (see text for details).

To simulate tamponade, we modeled graded increases in pericardial fluid (i.e., the reverse of the pericardiocentesis procedure in which fluid is removed in measured aliquots). Figure 2 is a plot of the steady-state diastolic chamber pressures and P_PERI _in response to increases of effusion volume. At V_PERI _of 800 ml, there is > 2 mmHg increase in P_PERI_. At 950 ml fluid accumulation, pulsus paradoxus is seen with an 11% variation in systolic blood pressure with respiration. At 1050 ml, all chamber pressures equilibrate within 2 mmHg of each other. We define a "chamber collapse index" as the mean percentage of a cardiac cycle in which P_PERI _exceeds chamber pressure, averaged over several cardiac cycles covering both the inspiratory and expiratory phases of respiration. At 1100 ml, RA collapse occurs over 34% of the cardiac cycle and LA over 20%.

**Figure 3:**
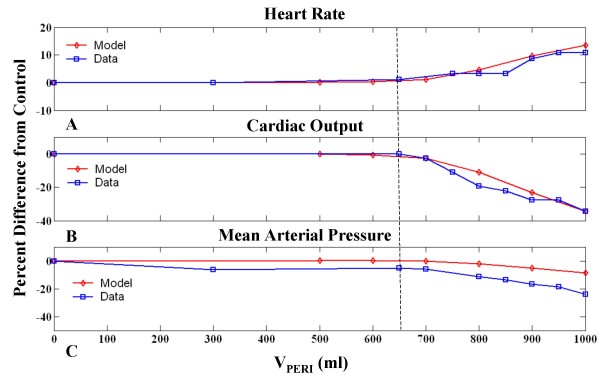
**Circulatory Indices as a Function of Pericardial Volume**. Percent change in circulatory indices as a function of pericardial volume (V_PERI_) for the model (diamonds) and patient data (squares) from [3]. Heart rate increases with V_PERI _up to 1000 ml (A), whereas cardiac output (B), and mean arterial pressure (C) decrease. Dotted line indicates point of significant deviation from control.

Above 700 ml, progressive increases in V_PERI _is accompanied by decreases in cardiac output (CO), mean arterial pressure (MAP), and associated activation of the baroreceptor reflex manifested as an increase in heart rate (HR). Figure 3 shows the percent change in these circulatory indices from the control state as a function of V_PERI_. Measured data points from the patient whose pericardium we have modeled [3] are shown for comparison. Figure 3 shows that the model provides good qualitative agreement with the measured hemodynamic indices HR and CO, however, the model is limited by a less satisfactory fit to MAP data. For all other model parameters to be operating in normal ranges, MAP behavior is compromised with a lesser drop with effusion than seen in data. The dotted line in Figure 3 indicates the point of significant percent change from control in all three indices which aligns well with data. As can be observed, MAP data at low effusion levels below the dotted line shows an unlikely drop that is different from the point of deviation in other indices, indicating the possibility of measurement error in the data of Reddy et al. Nonetheless, even with a correction in pressure offset, the model-generated rate of decline in MAP with increased pericardial effusion volume is lower than that seen in the data. Hence, the model provides only a qualitative fit to the patient data.

#### Right Heart Relationships in Tamponade

To examine the right heart hemodynamics in tamponade without overlying respiratory variations, P_PL _is set to the mean, thus simulating breath-holding. The atrium may be envisioned as a contractile storage chamber with an inflow from the vena cava compartment and an outflow through the tricuspid valve to the RV chamber. Diastole is defined as the interval between tricuspid valve opening and closure [19].

**Figure 4:**
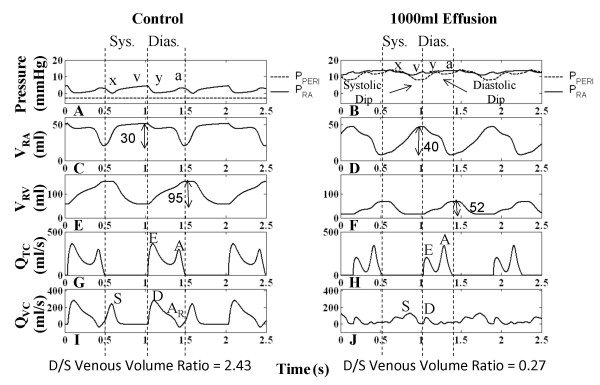
**Right Heart Hemodynamics**. Right heart hemodynamic waveforms for the control and 1000-ml effusion cases during apnea at mean pleural pressure (-3 mmHg). The systolic and diastolic intervals are indicated, with relatively shorter intervals in the 1000-ml effusion case due to higher heart rate. The left column shows normal pericardial pressure and right atrial pressure (Panel A), right atrial volume (Panel C), right ventricular volume (Panel E), tricuspid flow (Panel G), and inferior vena caval flow (Panel I), respectively. With 1000-ml effusion (right column), the right atrial pressure waveform is elevated to equalize pericardial pressure (Panel B) and the y-descent in particular is reduced (Panel B). Pericardial pressure displays two dips in pressure, corresponding to ventricular ejection (labeled systolic dip) and atrial ejection (labeled diastolic dip). Systolic atrial filling is slowed as shown by the gradual increase in right atrial volume (Panel D) and slower vena caval flow (Panel J). The reduced diastolic venous return (Panel J) is associated with a lower right atrial volume at end diastole (Panel D). Right ventricular volume variation exhibits reduction due to both filling and stroke output changes, with volume labels (ml) shown (Panels E-F). The E-wave is reduced and the A-wave is more prominent (Panel H). The reversed component of vena caval flow (A_R_) is no longer present (Panel J). The diastolic-to-systolic (D/S) venous volume ratio is shown below each case, which decreases with tamponade. See text for details.

Figure 4 shows that for the control case, RV systole begins after tricuspid valve closure and the RA continues to relax causing a reduction in RA pressure (P_RA_), i.e., the x-descent. Systolic filling of the RA consists of a fast and slow component as is seen in the RA volume (V_RA_) curve (Figure 4C) and in P_RA _v-wave (Figure 4A). The fast component of systolic RA filling is associated with the brisk systolic component (S) of vena caval volume flow Q_VC _(Figure 4I). In early diastole, the characteristic two-peak volume flow through the tricuspid valve (Q_TC_) (Figure 4G) equivalent to the more familiar Doppler transvalvular flow velocity measurements, corresponds to the onset of the y-descent in P_RA _(Figure 4A). In this communication, we describe features of transvalvular volume flow with the same terminology used in describing velocity measurements (i.e., E- and A-waves). Early diastole is marked by the prominent E-wave in Q_TC _(Figure 4G) and the beginning of diastolic (D) Q_VC _(Figure 4I). This is followed by a slow filling period (diastasis), and late in RV diastole, the RA chamber contracts contributing flow in both the forward direction (A-wave component of Q_TC _in Figure 4G) and the reverse direction (A_R _component of Q_VC _in Figure 4I). V_RA _reflects three diastolic flow stages that correspond to E-wave, diastasis, and A-wave of the Q_TC _(Figure 4C and 4G), with V_RA _reduction seen in the first and third stages. The relatively smaller first reduction reveals that Q_TC _> Q_VC_. The third stage reflects RA contraction reducing V_RA _(Figure 4C) and increasing P_RA _(a-wave in Figure 4A) to the extent that Q_VC _is reversed (A_R _component in Figure 4I), producing RA outflow in both directions. RV volume (V_RV_) in Figure 4E reflects the three-stage process of ventricular filling.

Examination of the P_PERI _waveform reveals key alterations during the cardiac cycle which may actively participate in the clinically observed features of tamponade. Under control conditions, P_PERI _is low relative to P_RA _and tracks the P_PL _(Figure 4A). It is important to recognize that when the total heart volume is constrained by the pericardial effusion, P_PERI _is now affected by changes in heart chamber volumes and becomes positive; it now tracks the diastolic RA pressure (Figure 4B) serving as the reference pressure for all heart chambers. Additionally, whereas P_PERI _is normally treated as a dependent variable at a given volume of pericardial effusion, as dictated by the P-V relationship of the pericardial space, because of the pressure transmission nature of the pericardial effusion, P_PERI _in tamponade assumes the role of an independent variable that actively modulates flows and pressures of other cardiac chambers. Specifically, changes in ventricular and atrial volumes are reflected in the P_PERI _waveform as two pressure dips attributed to ventricular and atrial ejection (systolic dip and diastolic dip, respectively) as observed in canine measurements [18,19]. We begin analysis of the pericardial constraint from the x-descent in P_RA _occurring in RV systole (Figure 4B). With tamponade, the x-descent is no longer related directly to relaxation of the RA. Rather, P_RA _is elevated and remains nearly constant by the pericardial constraint and the x-descent feature is delayed, decreased in magnitude and substantially slowed in its time course. At this point a prominent systolic dip in P_PERI _is seen, coincident with right and left ventricular ejection, which relieves the pericardial constraint on RA and allows venous return to refill the atrium (Figure 4B and 4J). P_RA _follows this decline in P_PERI _creating the delayed and slowed x-descent, and as the RA is allowed to slowly refill (Figure 4D), P_RA _separates from P_PERI _forming the v-wave. Thus, in contrast to the control condition, in which the x-descent precedes the RV ejection occurring during the isovolumic RV contraction and RA relaxation, the x-descent in tamponade is delayed and diminished in amplitude and occurs following the onset of RV ejection. Termination of the v-wave corresponds to maximum V_RA _and the minimum point in the systolic dip in the P_PERI _waveform. At tricuspid valve opening, there is a reduced RA-RV pressure gradient (reduced E-wave in Figure 4H) and a severely curtailed venous return flow (D component of Q_VC _in Figure 4J) as P_RA _is at its peak. V_RA _change results from a balance of Q_VC _(inflow to RA) and Q_TC _(outflow from RA) and the large decline in the D component of Q_VC _in tamponade is responsible for the smaller decrease in V_RA _during the early diastole phase. As the tricuspid E-wave declines, V_RA _continues to decline at a slower rate. When the RA contracts (a-wave feature in P_RA_), it produces a strong tricuspid flow (enhanced A-wave in Figure 4H) that reduces V_RA _to very low levels. Unlike control, there is no reversal in Q_VC _in severe tamponade. A comparison of the change in V_RA _and V_RV _during diastole can be made to infer the amount of vena cava inflow during diastole. For the control case, while V_RV _increases by 95 ml, V_RA _decreases only by 30 ml, indicating a significant simultaneous refilling of the RA during diastole (Figure 4C and 4E). In tamponade, the ventricular volume increases by 52 ml, while the atrial volume decreases by 40 ml, indicating little inflow from the vena cava (Figure 4D and 4F). Thus, most of the blood in the RA is transferred to the RV, with little refilling of the RA from the venous side in diastole. The pattern of diastolic increase in V_RV _also changes with tamponade, with smaller early filling, a period of very slow increase during diastasis, and a stronger increase coinciding with atrial ejection (compare Figure 4E and 4F). During these diastolic events, the y-descent feature is decreased substantially, reflected by a decreased E-wave, and P_RA _continues as an elevated, slowly increasing pressure (Figure 4B). In late ventricular diastole, a second smaller decline occurs in the P_PERI _waveform due to atrial ejection (diastolic dip), providing some relief from pericardial constraint. Subsequently, P_PERI _increases slowly due to a very limited diastolic venous return continuing into the systolic interval. This slow return delays the occurrence of the x-descent. Model measurements of common clinical indices are given in Table 1. With effusion, these clinical indices fall outside of normal range [11] signifying abnormal functionality.

**Table 1:** Model-Generated Common Clinical Indices

**V_PERI _(ml)**	**E/A Ratio**		**DT (sec)**		**IVRT (sec)**	
	
	*Right*	*Left*	*Right*	*Left*	*Right*	*Left*
15 (control)	1.2	1.2	0.235	0.190	1.110	0.082

1000 (severe tamponade)	0.6	0.5	0.120	0.089	0.198	0.082

Model measurements of common clinical indices in the right and left hearts for control (15 ml) and severe tamponade (1000 ml) effusion levels: E/A ratio, deceleration time (DT), and isovolumic relaxation time (IVRT).

#### Left Heart Relationships in Tamponade

**Figure 5:**
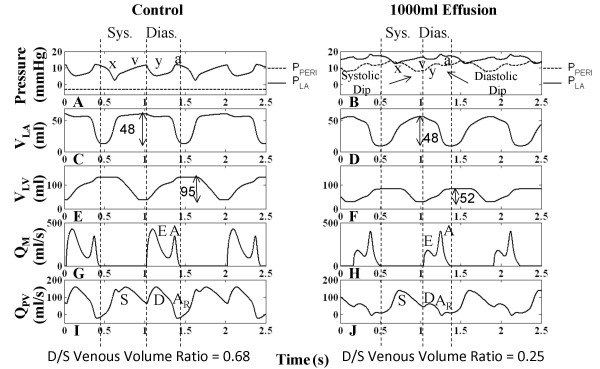
**Left Heart Hemodynamics**. Left heart hemodynamic waveforms for the control and 1000-ml effusion cases during apnea at mean pleural pressure (-3 mmHg). The systolic and diastolic intervals are indicated, with relatively shorter intervals in the 1000-ml effusion case due to higher heart rate. The left column shows normal pericardial pressure and left atrial pressure (Panel A), left atrial volume (Panel C), left ventricular volume (Panel E), mitral flow (Panel G), and distal pulmonary venous flow (Panel I), respectively. With 1000-ml effusion (right column), the left atrial pressure waveform is elevated (Panel B) with diminished atrial relaxation (x-descent) and diastolic ventricular filling (y-descent) (Panel B). Pericardial pressure displays two dips in pressure, corresponding to ventricular ejection (labeled systolic dip) and atrial ejection (labeled diastolic dip). Systolic atrial filling is slowed as shown by the gradual increase in left atrial volume (Panel D). Ventricular volume variation is reduced as a result of both reduced LV filing and ejection, as shown by the volume labels in Panels E-F. The E-wave is reduced and the A-wave is more prominent (Panel H). The diastolic (D) and reversed (A_R_) components of venous flow are diminished (Panel I). The diastolic-to-systolic (D/S) venous volume ratio is shown below each case, which decreases with tamponade. See text for details.

The left heart hemodynamics also reflects the compressive effects of pericardial effusion on LA volume (V_LA_) (compare Figure 5C and 5D) and diastolic LV volume (V_LV_) (compare Figure 5E and 5F). Here, diastole is defined as the interval between mitral valve opening and closure. Left atrial pressure (P_LA_) is elevated in tamponade (Figure 5B) compared to control (Figure 5A), with limited atrial relaxation (x-descent). The pericardial constraint slows systolic LA filling (compare Figure 5C and 5D), and the volume constraint imposed by P_PERI _limits diastolic pulmonary venous return shown in the distal venous flow waveform (compare Figure 5I and 5J). As in the right heart, transvalvular flow is altered with reduced early LV filling (compare Figure 5G and 5H). The corresponding diastolic y-descent in P_LA _is diminished (Figure 5B). Overall, the compressive effects of pericardial constraint are manifested to a lesser degree in the relatively thick-walled left heart with its slightly higher diastolic pressures.

The pre-ejection period for the LV (LPEP) is normally slightly longer than that for the RV (RPEP) as noted in [32] (compare Figure 4E and Figure 5E). This asynchrony in ventricular ejection times becomes much more pronounced in tamponade (discussed later) and plays a role in modifying the shape of the x-descent feature of the P_RA _waveform. The x-descent waveform is shaped by P_PERI _which has two components, the first corresponding to RV ejection and the second LV ejection. Figure 4F and Figure 5F indicate that the ventricles each eject 52 ml, however the end-diastolic filling volumes are quite different (68 ml V_RV _and 80 ml V_LV_) indicating that the RV is compressed to a much higher degree than the LV.

#### Atrioventricular Interaction

Examination of Figure 4J and Figure 5J shows that in severe tamponade, diastolic venous return is particularly decreased when compared to systolic venous return. Theoretically if diastolic venous return reaches zero, the only time the atrium can fill is during systole. At this stage, atrial filling is entirely conditional upon ventricular ejection, a term called maximum atrioventricular (AV) interaction [19]. Beloucif et al. [19] have quantified AV interaction in terms of a diastolic-to-systolic (D/S) venous return volume ratio. We obtained systolic and diastolic inflow volumes per beat by integrating venous volume over the systolic and diastolic time intervals, respectively. These intervals are denoted in Figure 4 and Figure 5, in which diastole is determined as the duration of ventricular filling, and systole the remainder of the cardiac cycle as in [19]. Calculation of venous return volumes indicated that in severe tamponade of 1000 ml effusion, diastolic vena cava return volume is reduced by 85% whereas the systolic volume actually increases by 40%. Thus, the right heart D/S ratio in venous return volume drops from 2.43 in control to 0.27 in tamponade (Table 2). In the left heart, diastolic pulmonary venous return volume is reduced by 73% and the systolic volume drops by 24%. The ratio of D/S pulmonary venous inflow volume also indicates a shift in the LA filling pattern in severe tamponade (1000 ml effusion) with a change in D/S ratio from 0.68 to 0.25 (Figure 5). The distal pulmonary venous flow waveform was used in this case analogous to the report by Beloucif et al. [19]. Table 2 shows diastolic and systolic venous return volumes for increasing levels of effusion. The shift toward systolic venous filling is apparent in the right heart (Figure 4I and 4J) with little change in maximum V_RA _at the end of the systolic interval, but a substantially decreased V_RA _at end-diastole related to a reduction in diastolic venous return. Diastolic left heart venous return volume has both reduced influx and a significantly reduced reversal flow (Figure 5J), which leaves V_LA _unaffected at end-diastole (compare Figure 5C and 5D). This dominant systolic atrial filling pattern is indicative of enhanced AV interaction primarily affecting the right heart consistent with the findings of Beloucif et al. [19].

**Table 2:** Diastolic and Systolic Venous Return Volumes with Pericardial Effusion

**V_PERI _(ml)**	**Right**			**Left**		
	
	*V*_*VC,D*_	*V*_*VC*,*S*_	*D/S Vol. Ratio*	*V*_*PV*,*D*_	*V*_*PV*,*S*_	*D/S Vol. Ratio*
*15 (control)*	55.0	22.6	2.43	37.5	55.4	0.68

*700*	48.8	24.4	2.00	33.5	54.7	0.61

*800*	37.0	27.4	1.35	26.0	53.0	0.49

*900*	20.7	30.7	0.67	16.6	48.5	0.34

*1000*	8.5	31.5	0.27	10.4	42.1	0.25

Venous return volumes during diastole and systole and the diastolic-to-systolic (D/S) volume ratios for increasing levels of effusion. For the right heart, vena caval return volume is given by V_VC,D _for diastole and V_VC,S _for systole. Similarly for the left heart, pulmonary venous return volume is given by V_PV,D _for diastole and V_PV,S _for systole. The D/S volume ratio decreases with increasing effusion.

#### Chamber Pressure-Volume Relationships

**Figure 6:**
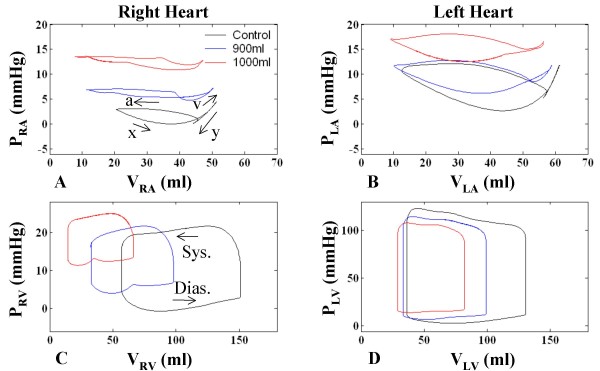
**Chamber Pressure-Volume Relationships**. Pressure-volume (P-V) relationships in the four chambers for control (no effusion), 900 ml effusion, and 1000 ml effusion. The RA pressure-volume loop is particularly altered with effusion, with a delayed and reduced x-descent, flattened y-descent, elevated pressure, and greater emptying. The left atrium displays similar characteristics to a lesser degree. P-V ventricular loops demonstrate chamber compression and show increasingly reduced stroke output and higher diastolic pressures. Atrial contraction causes a dip in pericardial pressure, drawing down RV pressure as well which causes a notching effect in the diastolic portion of the RV loop.

Figure 6 shows the P-V relationships for the four heart chambers at control, 900 ml effusion (mild tamponade), and 1000 ml effusion (severe tamponade). Breath-holding is simulated with P_PL _held at mean. In the control case for the RA, filling of the RA is coincident with RV systole, beginning at minimum V_RA _with the x-descent in P_RA _(see labeling on Figure 6A) and continuing smoothly into the v-wave of increasing P_RA _as V_RA _rises to a peak at the end of the RV systolic period (Figure 4A and 4C and Figure 6A). The RV diastolic period has three components, beginning with a sharp decline in P_RA _(y-descent; Figure 4A and Figure 6A) with a modest decline in V_RA_. This is followed by a period of diastasis, where pressure increases slightly as does V_RA _due to Q_VC_. Finally, atrial contraction ensues with increasing P_RA _and a relatively strong decrease in V_RA _(Figure 4A and 4C and Figure 6A). This completes the upper RV diastolic portion of the RA P-V loop, where diastole and systole are defined relative to the RV mechanical cycle. Time is implicit on these atrial P-V loops, increasing in a counterclockwise fashion over the cardiac cycle.

Atrial P-V loops show general movement upward and to the left, toward higher atrial pressures and lower minimum volumes (Figure 6A and 6B). This is especially true for the RA, where with progression of tamponade there is a steady decline in the minimum volume point on the loop. The maximum RA volume point also declines slightly with higher level of tamponade (compare maximum volume in Figure 4C and 4D). The flattened appearance of the RA loops of Figure 6A with minimum chamber volume reaching very low levels convey a powerful image of the constrictive effect of pericardial effusion on thin-walled heart chambers. The slope of the y-descent declines in the RA P-V domain (Figure 6A) with increasing tamponade, and the y-descent is followed by a slowly increasing pressure for the remainder of the RV diastolic interval (upper portion of the loop). A slow v-wave follows a delayed and reduced x-descent feature in the systolic portion of the RA P-V loop. P_RA _remains relatively constant over the latter portion of the RV systolic interval. V_RA _excursion is increased in tamponade relative to control. Progressive pericardial constraint is associated with elevation of P_LA _and flattening of atrial P-V loops (Figure 6B). With increasing effusion (Figure 6C and Figure 6D), the ventricles exhibit a rise in diastolic pressure and a reduction in volume and pressure excursion. In tamponade and during the ventricular filling phase, the complex changes in the P_PERI _waveform sculpt the diastolic P-V relationship including the notching effect observed in Figure 6C.

#### Section Summary

Graded increases in pericardial volume simulate tamponade hemodynamic changes both at the right and left heart. The right heart hemodynamic changes can be summarized as follows: 1) the pericardial pressure tracks the chamber pressures and not the pleural pressure; 2) RA filling is delayed and diminished such that the x-descent occurs after the onset of RV ejection, rather than at the onset of RV isovolumic contraction; 3) the early diastolic filling (E-wave) is diminished and the late filling (A-wave) assumes greater proportion, due to a markedly decreased vena cava D-component; 4) atrial filling is restricted significantly to ventricular systole, in contrast to the normal filling during both ventricular systole and diastole, leading to a diminished or absent y-descent. The left heart hymodynamics are altered in parallel. Informative findings of these changes in tamponade are well visualized with atrial and ventricular P-V loops. There is evidence >11% pulsus paradoxus and atrial collapse (34% RA, 20% LA). The CO and MAP are compromised and there is demonstration of baroreflex activation and tachycardia.

Importantly, the pericardial pressure waveform in tamponade reflects local volumetric changes in the heart chambers, in particular the diastolic and systolic dips in pressure, which in turn influence the filling capability of the chambers. As a result of this pericardial constraint, venous return to the atria is progressively higher in systole rather than in diastole, as the ventricles are contracted and the heart occupies less volume, producing atrioventricular interaction that largely determines the heart's filling volume.

### Effects of Respiration

During inspiration, there is an increase in venous return to the right atrium. Lowered intrathoracic pressure on inspiration lowers pressure in intrathoracic systemic veins, pericardium, and cardiac chambers. Consequently, flow from extrathoracic systemic veins is increased and more blood flows to the right heart. This augmented blood flow appears in the left heart two to three beats later (during expiration for a person at rest), i.e., the "transit time" for blood to travel through the pulmonary vasculature [16,17]. In severe tamponade, the high P_PERI _due to effusion creates a competition for filling space, which increases interaction between the ventricles. During the inspiratory increase in systemic venous return, filling of the left heart is compromised, lowering LVSV and aortic pressure [3,6,17,33]. Alternately, during expiration, left heart filling is favored over the right heart. The resulting variation in LVSV can cause more than a 10% variation in arterial pressure with inspiration, or pulsus paradoxus [3]. In our model, the critical pericardial effusion volume for production of pulsus paradoxus at normal breathing levels is 950 ml.

**Figure 7:**
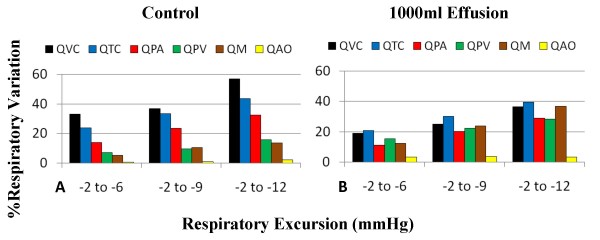
**Flow Variation with Different Respiratory Excursions**. Percent variation between inspiratory and expiratory flows for different breathing waveform excursions (e.g., -2 to -6 mmHg) for control (Panel A) and tamponade (Panel B). As respiratory excursion increases, respiratory variation increases for all flows. Under control conditions, respiratory variation is significantly higher in the right heart than in the left. However, with severe tamponade, the level of respiratory variation in the left heart increases, becoming more comparable to that of the right heart. (QVC = vena cava flow; QTC = tricuspid flow; QPA = pulmonary artery flow; QM = mitral flow; QAO = aortic flow).

To analyze the effect of respiration on hemodynamics, three sinusoidal breathing patterns (with modulation of depth and excursion) were used in the model and the percentage variation between expiration and inspiration for inlet, outlet, and transvalvular flows was calculated. Figure 7 shows the different levels of respiration and associated percent variation. With greater excursion and lower P_PL _on inspiration, overall respiratory variations increase. In the control case (Figure 7A), right heart flows (Q_VC_, Q_TC_, Q_PA_) have much greater respiratory variation than the left (Q_PV_, Q_M_, Q_AO_). For example, at different levels of respiratory effort, the flow variations at the tricuspid valve (Q_TC_) range from 23–43%, whereas the flow variations at the mitral valve (Q_M_) range from 5–13%. With severe tamponade, the flow variations at the tricuspid valve range from 21–40%, but the flow variations at the mitral valve increase its range to 12–37% (Figure 7B). The increased flow variation at the left heart has been used as a clinical index for hemodynamic important pericardial effusion or cardiac tamponade [1,34]. These comparable levels of respiratory variation on the right and left sides are strong indicators of increased ventricular interaction, as discussed later.

#### Pulsus Paradoxus

**Figure 8:**
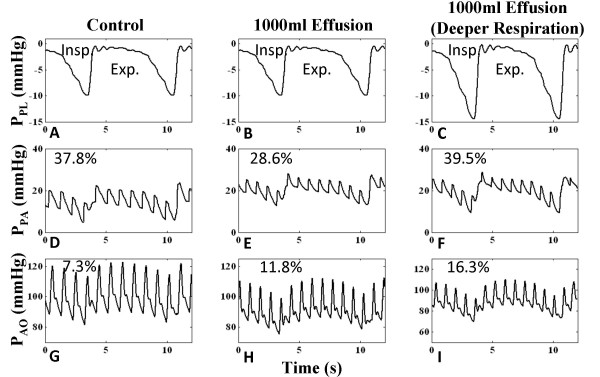
**Arterial Pressure Respiratory Variation**. Model-generated distal arterial pressure for the right (pulmonary arterial pressure (P_PA_)) and left (aortic pressure (P_AO_)) heart. In the control case for the left heart with respiratory excursion of -1 to -10 mmHg, 7.3% variation exists (Panel G). With the same breathing pattern, aortic pressure drops and an 11.8% pressure variation exists (Panel H), indicating pulsus paradoxus. For the right heart, pressure elevates and variation decreases with effusion (compare Panels D-E). With a deeper breathing pattern (-1 to -15 mmHg), variation increases in both cases (Panels F and I).

Figure 8 shows the presence of pulsus paradoxus with effusion. The control case demonstrates 7.3% distal aortic pressure variation with respiration level -1 to -10 mmHg (Figure 8G). Effusion increases pulse pressure variation to 11.8% (Figure 8H), but the depth of breathing influences the level of variation, as shown by an increased variation of 16.3% with deeper inspiration to -15 mmHg (Figure 8I). With severe tamponade (1000 ml effusion), pulmonary arterial pressure (P_PA_) is increased and shows less pressure excursion due to the increase in pulmonary blood pooling (discussed below), but P_AO _is decreased due to the decline in cardiac output and the respiratory variation increases. Hence, there are opposing effects with pericardial constraint on the pulmonary and aortic pressures. However, increased respiratory variation increases pressure variation at both sides (Figure 8F and 8I).

#### Interventricular Septum

Septal motion in tamponade has been studied in an effort to suggest mechanisms underlying abnormal hemodynamics and respiratory variation [18]. We first present the septal model followed by our model results with regard to septal contribution in tamponade.

##### Septal Model

The septal model we have employed encompasses septal motion for the complete cardiac cycle, mimicking biphasic motion as noted by others [13,14]. As detailed in our own studies [12,35], a storage compartment of volume V_SPT _is defined as the volume bound by the current septal position and its unstressed position (Figure 1A), where positive and negative V_SPT _indicate rightward and leftward septal curvature, respectively. LV volume is therefore defined as the volume bound by the LV free wall and the unstressed septum plus V_SPT_, and RV volume is defined as the volume bound by the RV free wall and the unstressed septum minus V_SPT_. The transmural pressure P_SPT _is defined as the difference between P_LV _and P_RV_. There is a systolic and diastolic phase in the pressure-volume (P-V) relationship for the septal compliant compartment, linear in systole, nonlinear in diastole. Secondly, the septum undergoes active contraction synchronized with RV and LV free wall contraction in systole, behaving as a third pump. Septal activation and the trans-septal pressure gradient both shape septal motion.

**Figure 9:**
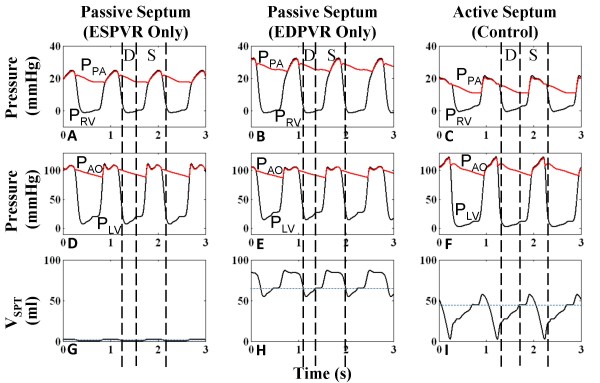
**Comparison of Septal Models**. Ventricular and arterial pressures and septal volume for three septal models – case a: passive septum with linear end-systolic pressure-volume relationship (ESPVR) held throughout cardiac cycle (Panels A, D, G); case b: passive septum with nonlinear end-diastolic pressure-volume relationship (EDPVR) held throughout cardiac cycle (Panels B, E, H); case c: active septum with linear ESPVR and nonlinear EDPVR modulated by a septal activation function in the cardiac cycle (Panels C, F, I) (see text for details). For case a, the septum is highly noncompliant and nearly fixed at neutral position. This curtails systolic P_LV _and creates an abnormal upward slope in systolic P_RV_. Case b severely bows the septum rightward and the relatively stiff septum, whose movement is subject only to left-to-right trans-septal gradient, mirrors P_LV_. Systolic P_RV _is high due to rightward septal position during systole. With the active septum of case c, systolic ventricular pressures have opposing slopes as seen in clinical data [11]. The septum is activated at systole to produce a strong leftward thrust (D = Diastole, S = Systole).

We find that a biphasic definition of the septal P-V relationship controlled by a septal activation function in a cardiac cycle is essential to accurately model a normal LV and RV pressure profile. Three septal models were simulated: a) a linear P-V relationship as observed in end-systole [36] and held throughout cardiac cycle – this models a stiff septum such as an akinetic septum b) a nonlinear P-V relationship applicable to end-diastole [35] and held throughout cardiac cycle – this models a compliant membrane such as a septal aneurysm c) a linear P-V relationship in end-systole and nonlinear P-V relationship in end-diastole and a combination of the two for the remaining cardiac cycle determined by a time-dependent activation function [35] – the current active septal model. Cases a and b are passive septal models, with V_SPT _independent of time, whereas case c treats the septum as an active pump synchronous to the active RV and LV free wall pumps. Figure 9 shows ventricular pressures and V_SPT _for the three cases. With passive septum case a, the septum is highly non-compliant and approximately fixed at neutral position (Figure 9G). Systolic behavior in P_RV _and P_LV _is divergent to that observed experimentally [11,33], with an upward slope in P_RV _(Figure 9A) and a distorted P_LV _(Figure 9D). Similarly with the passive septum of case b, the septum is strongly bowed right, and its movement is shaped by the left-to-right trans-septal gradient, thereby mirroring the shape of P_LV_(Figure 9H). Systolic P_RV _is sloped upward, higher than normal (Figure 9B), systolic P_LV _is flattened and diastolic P_LV _is heightened (Figure 9E). The active septum of case c displays the opposing slopes in systolic ventricular pressures as seen in canine [37] and clinical data [11] (Figure 9C and 9F), and a large septal leftward thrust as observed experimentally [13,14] to the near-neutral position is seen in systole (Figure 9I). The septum moves slowly rightward in diastole, coincident with increasing left-to-right pressure gradient, and when free wall contraction commences, the septum also begins to contract pushing further into the right ventricle before the leftward thrust. "Septal priming" prior to LV ejection initiates RV outflow movement and a lengthened RV ejection is observed. At the end of systole, the septum recoils toward the RV giving an extra boost to RV stroke output, before pulmonic valve closure. Thus pulmonic valve closure is delayed by an active septum and septal assistance to RV systolic function [37] can be pinpointed to these two occurrences, both of which are not present in cases a and b, which may be hemodynamically significant.

**Figure 10:**
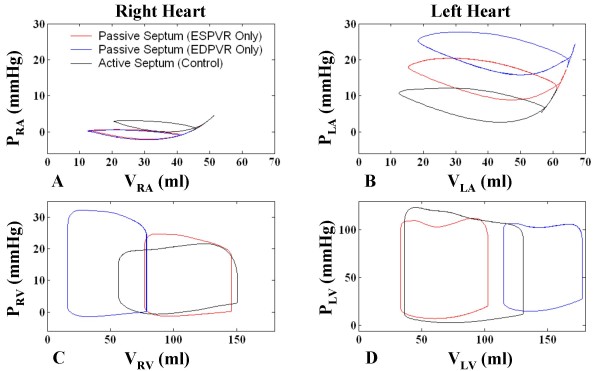
**Chamber Pressure-Volume Relationships using Different Septal Models**. P-V relationships for the four cardiac chambers shown for three septal model cases – case a: passive septum with ESPVR only (red); case b: passive septum with EDPVR only (blue); case c: active septum with ESPVR and EDPVR modulated by an activation function (see text for details). For case a, the stiff, unstressed septum reduces V_LV_, increasing end-diastolic P_LV _and restricting LV filling, while increasing systolic P_RV _due to no leftward movement, and not contributing to RV ejection. In case b, the less-compliant, rightward-shifted septum expands V_LV _and reduces V_RV _overall, increasing systolic P_RV _and contributing little to ventricular ejection (Panels C-D). In both cases, the reduced cardiac output decreases P_RA _(Panel A) and end-diastolic P_RV _(Panel C). Reduced stroke volume causes blood to accumulate in the pulmonary bed, increasing P_LA _(Panel B).

P-V loops of the four cardiac chambers are given in Figure 10 for all three cases. For case a, the stiff septum in the neutral position reduces the size of LV (Figure 10D), increasing end-diastolic P_LV _and restricting LV filling, while increasing systolic P_RV _due to no leftward movement, and providing no aid to RV ejection (Figure 10C). Alternately in case b, the relatively stiff and severely rightward-bowed septum expands V_LV _and reduces V_RV _overall, greatly increasing systolic P_RV _and playing a limited role in ventricular ejection (Figure 10C–D). In both cases, the reduced cardiac output decreases P_RA _(Figure 10A) and end-diastolic P_RV _(Figure 10C). On the other hand, uncirculated blood accumulates in the pulmonary vasculature increasing P_LA _(Figure 10B). Increased RV stroke volume of case c over cases a and b is clearly demonstrated by the P-V loops analysis.

##### Septal Motion in Tamponade

**Figure 11:**
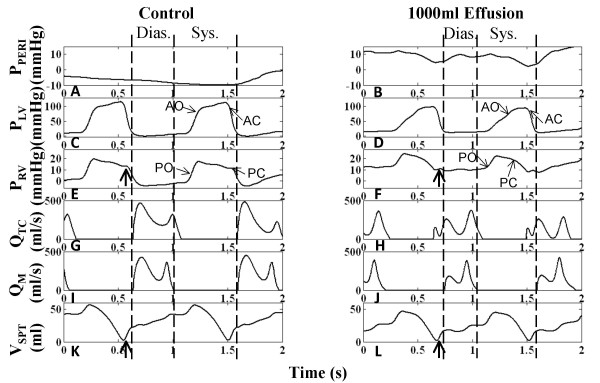
**Relation of Septal Motion to Hemodynamics**. Septal movement is tracked by plotting septal volume V_SPT _vs. time, in which positive V_SPT _is rightward septal position, zero V_SPT _is the unstressed or neutral septal position, and negative V_SPT _is leftward septal position. Pericardial pressure shown in control (Panel A) and 1000 ml effusion cases (Panel B) during inspiration. Remaining panels show LV pressure (Panels C-D), RV pressure (Panels E-F), tricuspid flow (Panels G-H), mitral flow (Panels I-J), and septal volume (Panels K-L). LV systolic and diastolic intervals are indicated by dashed vertical lines, coincident with mitral valve opening and closure. The ejection times are offset in the two ventricles, with late aortic valve opening (AO) coincident with delayed septal leftward thrust and early pulmonic valve closure (PC) with premature reduction in P_RV _due to P_PERI _systolic dip. The septum remains left-shifted at the start of right ventricular filling. With rightward septal movement upon deactivation (bold arrows in Panels E-F and K-L) flow is interrupted and a split E-wave is produced (see text for details). (AO = aortic valve opening, AC = aortic valve closure, PO = pulmonic valve opening, PC = pulmonic valve closure)

As mentioned previously, the active septum (case c in the previous section) has been employed consistently in this modeling study. To better analyze septal contribution to tamponade, plots of septal volume (V_SPT_) can be used to track septal movement, where septal volume is defined as the volume offset from the neutral septal position (see Figure 1B), in which positive V_SPT _indicates a rightward-shifted septum, and negative V_SPT _indicates a leftward shift. Due to the overall left-to-right trans-septal pressure gradient, the healthy septum is bowed rightward and V_SPT _is always positive. Septal volume is shown in Figure 11K–L for control and 1000-ml effusion cases, respectively. In early systole, the left-to-right pressure gradient produces an initial rightward septal movement (Figure 11K). Septal activation synchronous with ventricular free wall activation and subsequent isovolumic contraction during the pre-ejection period (PEP) produces leftward movement against the gradient which continues when the aortic valve opens (see label on Figure 11A). With aortic valve closure and septal deactivation synchronous with free wall deactivation, there is a left-to-right trans-septal pressure gradient producing the characteristic opposing slopes in systolic P_RV _and P_LV _(see Figure 11C and 11E), moving the septum rightward. This movement serves to enhance RV stroke volume [37] and consequently improve LV filling through greater LV filling space. The onset of diastole is signaled by the opening of the tricuspid and mitral valves (Figure 11G and 11H) and the septum remains passive throughout diastole moved by the much smaller trans-septal pressure gradients set up by the early and late inlet flows to both ventricles [12]. P_PERI _(Figure 11A), tracking the low-amplitude intrathoracic pressure, has no significant influence in shaping cardiac pressures within the cardiac cycle. In severe tamponade however, P_PERI _bears a cardiac variation (Figure 11B) controlling chamber pressures, particularly those of the more compliant right heart. P_PERI _undergoes a systolic dip in pressure (see label on Figure 4B) drawing down P_RV _prematurely and closing the pulmonic valve (Figure 11F). Meanwhile, P_LV _displays a prolonged PEP that is associated with a delayed aortic valve opening (Figure 11D). Systolic ejection is further desynchronized in the two ventricles as compared to the control. The septum moves rightward due to the initial systolic left-to-right trans-septal gradient (Figure 11L). This is followed by PEP with associated leftward septal movement. There movement shows a reduced downward slope in concert with an increased isovolumic period (compare Figure 5E with F, and Figure 11C with D). On ejection, the septum actively supports the LV stroke output with a leftward movement. The mid-systolic decline in P_RV _is associated with an early positive atrioventricular gradient across the tricuspid valve with resultant early inlet flow (Figure 11H), while the septum still remains left-shifted (Figure 11L). Therefore the tricuspid and mitral transvalvular flow initiations are also desynchronized. Comparing the late systolic ejection period, the right septal movement under normal situation occurs before the pulmonic valve closure, thus the septal movement has a functional component in assisting RV output (arrows in Figure 11E and 11K) [37]. In contrast, in tamponade, the right septal movement (Figure 11L) in late systolic cycle occurs at a time when the pulmonic valve has already been closed. Thus the septal movement contributes nothing to RV outflow in tamponade. Rather, it causes a brief increase in P_RV _(arrows in Figure 11F and 11L), which reduces the AV gradient and interrupts RV filling. This interruption of early diastolic Q_TC _is observed as a split E-wave (Figure 11H). Comparing Figure 11K and 11L, diastolic septal volume is reduced, indicating an overall leftward shift in septal position.

**Figure 12:**
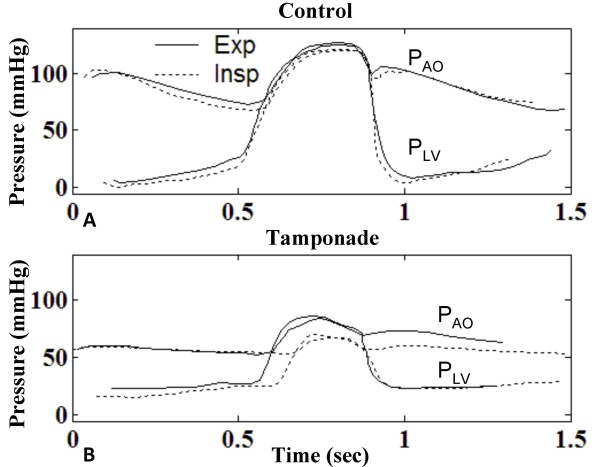
**Digitized Recordings of Left Ventricular Variation in Respiratory Cycle**. High-fidelity micromanometer recordings of left ventricular (P_LV _and aortic pressure (P_AO_) before and after pericardiocentesis, digitized from clinical data published in [33]. Control (Panel A) is assumed to be post-pericardiocentesis. In both panels, a single cardiac cycle during expiration has been overlaid with a single cardiac cycle during inspiration, aligned at aortic valve closure for comparison. In the control case (Panel A), pressures drop slightly on inspiration. With tamponade (Panel B) pressures fall significantly in systole, pre-ejection period is delayed in cardiac cycle, and ejection time is reduced. Data from Murgo et al. [33], specifically, Figs. 2–3, pp. 193–4.

In severe tamponade, the abnormal prolongation of PEP in P_LV _bears a respiratory variation that can be observed in both canine [18] and clinical data [33,38]. Figure 12 shows digitized record of analog data reported in a cardiac tamponade case by Murgo et al. [33], where plots of P_LV _and aortic root pressure (P_AO_) during expiration (solid line) and inspiration (dashed line) are overlaid for the two cases post-pericardiocentesis (control) and pre-pericardiocentesis (1000 ml effusion). We note from this data that the isovolumic relaxation phase remains unchanged throughout the respiratory cycle; hence we have aligned single cycles of P_LV _in inspiration and expiration relative to this phase and in particular with aortic valve (AOV) closure. In control, respiratory effects are minor as reported in [38]. With tamponade (Figure 12B) pressures fall significantly in systole, PEP is slowed, and ejection time is reduced (see Table 3).

**Table 3:** Model-Generated Ejection Time and Volume Indices During Various Phases of the Respiratory Cycle

**P_PL _(mmHg)**	**Control** **(Cardiac Period = 0.96 sec)**								**1000 ml Effusion** **(Cardiac Period = 0.84 sec)**							
	
	*RVET (sec)*	*RVEDV (ml)*	*RVSV (ml)*	*RPEP (sec)*	*LVET (sec)*	*LVEDV (ml)*	*LVSV (ml)*	*LPEP(sec)*	*RVET (sec)*	*RVEDV (ml)*	*RVSV (ml)*	*RPEP (sec)*	*LVET (sec)*	*LVEDV (ml)*	*LVSV (ml)*	*LPEP (sec)*
*Exp. (-1)*	0.37	133.2	77.7	0.12	0.29	134.1	98.3	0.21	0.23	54.3	42.0	0.11	0.25	92.9	64.6	0.20

*Insp. (-10)*	0.38	168.4	108.3	0.08	0.28	126.5	90.8	0.23	0.26	80.7	66.6	0.04	0.18	75.3	48.0	0.28

*% Diff*.	2.7	26.4	39.4	-33.3	-3.5	-6.0	-7.6	9.5	13.0	48.6	58.6	-63.6	-28.0	-19.0	-25.7	40.0

RV and LV ejection time (RVET and LVET), RV and LV end-diastolic volume (RVEDV and LVEDV), RV and LV stroke volume (RVSV and LVSV), and RV and LV pre-ejection period (RPEP and LPEP) for expiration and inspiration. Percent difference with respect to expiration is given. Respiratory variation is increased with tamponade, with reduced ejection time, end-diastolic volume, and stroke volume and increased pre-ejection period on inspiration seen in the left heart, but opposite effects in the right heart.

**Figure 13:**
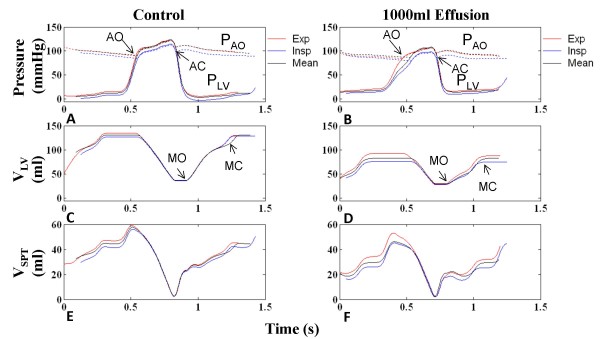
**Left Ventricular Performance Variation in Respiratory Cycle**. An in-depth look at respiratory variation in left ventricular performance with tamponade. Model results are given for the control case and the severe tamponade case (1000 ml effusion) in the first and second columns, respectively. One cardiac cycle displaying P_LV _and P_AO _during expiration (red), inspiration (blue), and fixed mean P_PL _(black) are overlaid with alignment of aortic valve closure (Panels A-B). Panels C-D show left ventricular volume, and Panels E-F show septal movement. In the control case, aortic valve opening (AO) and pre-ejection period (PEP) varies with respiration; specifically, during inspiration, PEP is slowed, delaying AO, and reducing the ejection time (Panel A). With tamponade, PEP on the whole is slowed compared to control, and the respiratory variation is exaggerated (Panel B). The elongated PEP is also evident in V_LV _(Panel D). During the PEP, the septum hangs in the neutral position, delaying the leftward thrust which can only start at AO (Panel F). (AO = aortic valve opening, AC = aortic valve closure, MO = mitral valve opening, MC = mitral valve closure)

Model-generated results reveal a similar respiratory effect on P_LV _with tamponade (Figure 13). Figure 13 relates P_LV_, V_LV _and septal volume under conditions of control and severe tamponade. The influence of respiration on these waveforms is shown in terms of single cycles of overlaid tracings during expiration, inspiration and breath-holding at mean P_PL_. Minor respiratory variations in LV end-diastolic volume (LVEDV) and diastolic V_SPT _are apparent in Figure 13C and 13E, with maximum V_LV _and septal rightward shift during expiration. Systolic respiratory variation is negligible. For the tamponade case, with inspiration, PEP is prolonged (Figure 13B) and aortic valve opening (AO) is delayed. Reduction in magnitude of LVEDV and its increased respiratory variation is evident (compare Figure 13C and 13D). The upstroke phase in P_LV _is correspondingly marked by a delayed leftward septal movement (Figure 13F), as V_SPT _bears a reduced downward slope until AO, when septal movement can once again support ejection with strong leftward movement.

With pericardial effusion, the combined effect of prolonged PEP and increase in heart rate shortens the LV ejection time (LVET) as noted in [38]. LVET is greatest in expiration and lowest in inspiration as evident in Figure 11, with a respiratory variation of 3% in control and 28% in severe tamponade (Table 3). Prior to ejection, LV filling volume varies as shown by respiratory variation in LVEDV of 6% in control and 19% in tamponade. As a result of both of these effects, LV stroke volume (LVSV) on inspiration is reduced by 5% in control and 26% in tamponade compared to expiration.

Respiratory variation occurs in RV ejection time (RVET) as well, but with an increase in RVET of 3% for control and 13% for tamponade on inspiration compared to expiration. RV end-diastolic volume (RVEDV) varies by 26% for control and 49% for tamponade. RV stroke volume (RVSV) also varies by 39% for control and 59% for tamponade with maximum RVSV at inspiration. As can be noted to occur with tamponade, ejection time is more varied in the LV than the RV [32], but end-diastolic volume varies more in the right. The combined effect of both gives a variation in stroke volume that is greater in the right. Results are tabulated in Table 3.

With tamponade, septal motion reflects abnormal hemodynamics associated with the pericardial constraint, i.e., the asynchrony and shortening of RV and LV ejection times which is exacerbated with inspiration. It should be noted that the use of term asynchrony to describe different ejection intervals should not be confused with asynchrony of electrical conduction through ventricles, as ventricular activation functions in the model are synchronized in time.

#### Pulmonary Vasculature

**Figure 14:**
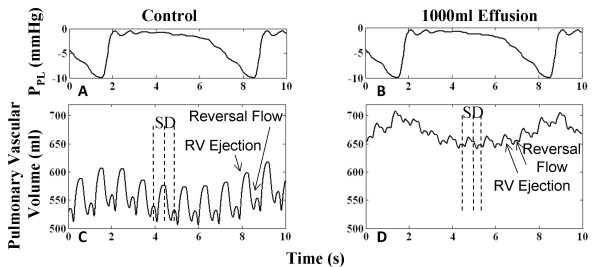
**Pulmonary Vascular Volume**. Pulmonary vascular volume for control (Panel B) and tamponade (Panel D) cases. With 1000 ml effusion, the mean pulmonary vascular volume increases by 20.8% due to compressed ventricles. Two increases in pulmonary volume take place in a cardiac cycle, namely, one due to RV ejection in systole (labeled S) and the other due to pulmonary venous reversal flow at end of diastole (labeled D) from the left atrium. Effusion limits pulmonary venous return, resulting in lower volume excursion per cardiac cycle and accumulation of blood in the pulmonary vasculature. Left heart atrioventricular interaction is observed with distinct systolic and diastolic venous return phases, the diastolic/systolic volume ratio smaller than in control. With respiration shown in Panels A-B, mean pulmonary blood is maximum on inspiration due to higher RV inflow and lower LA outflow.

The pulmonary vasculature serves as a blood reservoir connecting the left and right hearts. In severe tamponade, pulmonary blood pooling is observed by a 20% increase in mean pulmonary vascular volume (compare Figure 14C and 14D). In the control scenario (Figure 14C), pulmonary volume displays two peaks in a cardiac cycle, the upward stroke in the major peak associated with RV systolic ejection, followed by a second upward stroke and minor peak associated with pulmonary venous reversal flow from the left atrium at the end of diastole [39]. In tamponade, this pattern is seen with reduced excursion in volume (Figure 14D), as a result of reduced RV stroke volume and elevated left heart pressures due to pericardial constraint. Left heart compression prevents normal venous return, possibly correlated with the accumulation of blood in the pulmonary vasculature. This is particularly true in inspiration as seen with the corresponding P_PL _waveforms (Figure 14A–B). Pulmonary venous return occurring during the downward stroke of the major peak exhibits a distinct two-phase return, i.e., systolic and diastolic return. Left heart AV interaction is evident with this feature, with increasingly prominent systolic return and diminished diastolic return.

#### Gas Exchange

**Figure 15:**
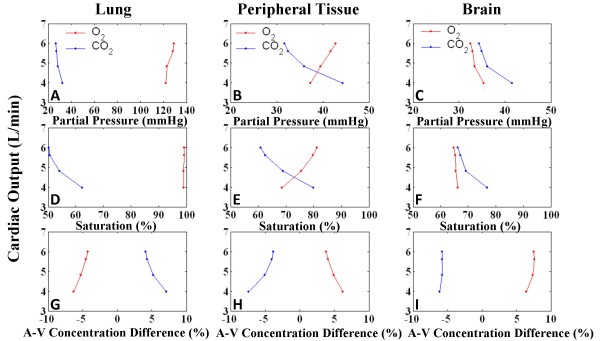
**Gas Exchange**. Gas exchange indices for lung, peripheral tissue and brain for graded levels of effusion. Panels A-C show cardiac output (CO) vs. partial pressures, Panels D-F show CO vs. percent saturation, and Panels G-I show CO vs. arterio-venous (A-V) percent concentration difference for O_2 _and CO_2_. With decreasing CO, peripheral tissue shows hypercapnia and decrease in oxygenation with lowering PCO2, percent saturation and increased CO_2 _A-V difference; opposite trends are present for O_2_. Lung displays similar characteristics to a lesser extent. The cerebral tissue shows signs of hypercapnia but autoregulation of cerebral blood flow limits hypercapnia and increases oxygenation, shown by minimal CO_2 _A-V difference, PO_2 _and SO_2_.

Model-generated acid-base balance in the lungs and alveoli, peripheral tissue, and brain were monitored for changes due to tamponade. For increasing levels of effusion, cardiac output was plotted against O_2 _and CO_2 _partial pressures, percent saturation, and arterio-venous (A-V) percent concentration differences (Figure 15), averaged over a respiratory cycle, modeled after a canine study [40]. Partial pressures were taken at the exit end of the tissue, i.e., venous pressures in peripheral tissue and brain, and arterial end of lung. Table 4 shows numerical values for key indices for control and severe tamponade. With tamponade, peripheral tissue reflects reduced blood oxygenation with lowered venous PO_2_, increased venous PCO_2_, and reduced SO_2 _(Figure 15B and 15E). Furthermore there is increased A-V difference in CO_2_. Situations of lowered cardiac output have been shown to exhibit increased A-V difference in CO_2 _[41-43] as well as increased venous PCO_2 _[41,42] due to a combination of arterial hypocapnia and venous hypercapnia. This is a result of slower blood flow causing accumulation of CO_2 _[44]. Reduced cardiac output causes a state of hypoperfusion, lowering O_2 _delivery to tissue demonstrated by PO_2 _and SO_2 _reduction. The lung tissue reflects these changes to a lesser extent. The cerebral circuit model on the other hand is autoregulated to maintain cerebral blood flow [26]. With hypercapnia, cerebral blood flow increases to improve oxygenation and increase CO_2 _washout, as shown by the maintenance of A-V difference in CO_2 _as well as PO_2_, SO_2_, and O_2 _A-V difference. Control values for partial pressure (Table 4) vary from previously reported values [11] as a different respiratory pattern has been used in this study. The lower inspiratory P_PL _causes more gas intake and the relatively longer expiratory phase releases higher levels of CO_2_. The simulation results suggest that in severe tamponade a deeper respiratory pattern would tend to counteract the hypoxic effect of lowered cardiac output with greater oxygenation and CO_2 _removal. Further investigation of breathing patterns seen in tamponade patients is suggested by these model observations.

**Table 4:** Model Parameters of Gas Exchange Function

**Tissue Type**	**Gas Exchange Parameter**	**15 ml effusion** **(control)**	**1000 ml effusion**
*Lung*	*PO_2 _(mmHg)*	*123.4*	*122.0*
	
	*PCO_2 _(mmHg)*	*33.9*	*32.4*
	
	*SO_2 _(%)*	*98.9*	*99.0*
	
	*A-V O_2 _concentration difference (%)*	*-4.0*	*-6.3*
	
	*A-V CO_2 _concentration difference (%)*	*3.9*	*7.1*

*Peripheral Tissue*	*PO_2 _(mmHg)*	46.1	37.3
	
	*PCO_2 _(mmHg)*	40.6	44.3
	
	*SO_2 _(%)*	81.1	68.6
	
	*A-V O_2 _concentration difference (%)*	3.7	6.2
	
	*A-V CO_2 _concentration difference (%)*	-4.5	-7.2

*Brain*	*PO_2 _(mmHg)*	35.4	35.4
	
	*PCO_2 _(mmHg)*	43.2	41.5
	
	*SO_2 _(%)*	65.5	66.2
	
	*A-V O_2 _concentration difference (%)*	6.7	6.3
	
	*A-V CO_2 _concentration difference (%)*	-6.5	-5.8

Model parameters indicating gas exchange function in lung, peripheral tissue, and brain. The breathing pattern is the pseudo-human respiratory waveform used in this study. With tamponade, peripheral tissue reflects effusion-induced oxygenation reduction with fall in O_2 _partial pressure (PO_2_), rise in CO_2 _partial pressure (PCO_2_), and reduced O_2 _percent saturation (SO_2_). There is little change in PO_2_, PCO_2 _and SO_2 _in the lung and brain. A-V O_2_/CO_2 _concentration difference measures the percent gas concentration difference in the arterio-venous (A-V) path. This O_2 _and CO_2 _concentration difference increases in lung and peripheral tissue with slower blood flow allowing more time for oxygen intake and increase in CO_2_. A-V differences remain largely unchanged in the brain due to the built-in autoregulation mechanism of the cerebral circuit. Using the pseudo-human respiratory waveform, control PO_2 _is higher and PCO_2 _is lower than previously reported model results [11] due to deeper inspiration and relatively longer expiration. SO_2 _is also slightly increased.

#### Section Summary

Respiration produces variation in cardiac pressures and flows. While in the control case respiration predominantly affects the right heart, with pericardial effusion, left heart respiratory variation becomes more significant and comparable to right heart respiratory variation. Pulsus paradoxus is present with 1000 ml effusion, with systolic blood pressure variation increasing further with deeper inspiration.

Modeling the biphasic motion of the septum, in which the septum actively recoils rightward and thrusts leftward during systole, is possible only with an active septal model, as demonstrated by comparison to two passive septum models. Motion of the active septum demonstrates the mechanisms for prolonged LPEP, shortened ejection times, and right-left asynchrony of filling and ejection periods.

The pulmonary vasculature demonstrates blood volume congestion in tamponade, in association with the elevated chamber pressures, and decreased equilibrium cardiac output.

Demonstration of gas exchange variation with pericardial effusion yields observations consistent with literature on cardiac failure revealing the state of hypoperfusion in tamponade.

### Mechanism of Pulsus Paradoxus

Shabetai, et al. [6,17] concluded that the mechanistic explanation for the inspiratory LV stroke volume (LVSV) drop in pulsus paradoxus is the increase in right heart venous return. We conducted two virtual experiments using the H-CRS model to examine this hypothesis, namely that right heart venous return is the primary cause for a change in LVSV. Two possible types of right-left interaction (parallel and series ventricular interaction) are demonstrated and their relative contributions to pulsus paradoxus are assessed.

#### Experiment 1: Effect of Increased Right Heart Venous Return

A virtual experiment based on the canine study of Shabetai et al. [17] was performed to observe the effect of increased right heart venous return on the hemodynamics of the left heart independent of respiration. Specifically, at a constant P_PL _of -3 mmHg, a triangular volume pulse of venous return to the right atrium mimicking a single inspiratory increase in venous return was introduced in the model and tracked over several heart cycles. In the control case, the volume pulse caused a 4-mmHg increase in pulmonary arterial pressure followed three beats later by a 10-mmHg increase in aortic pressure. However, under conditions of severe tamponade, the same volume pulse caused a 5-mmHg increase in pulmonary arterial pressure and a simultaneous 5-mmHg drop in aortic pressure, followed by a 9-mmHg increase from baseline in aortic pressure three beats later. The stroke volume variation demonstrated takes place over a period of roughly 7 seconds, or the duration of a single breathing cycle, and the arterial pressure excursion matches that seen in a single breath. This experiment demonstrates two effects of increased venous return that are exaggerated in tamponade: the immediate drop in LVSV as well as an increase in LVSV occurring three beats later.

#### Experiment 2: Isolated Series and Parallel Ventricular Interactions

The second experiment involved isolation of the two ventricular interaction mechanisms for analysis of their relative contributions to LVSV variation. To study series interaction alone, the septum was stiffened 100× from control to eliminate parallel ventricular interaction via the septum. Secondly, P_PERI _was held at mean pressure to eliminate parallel interaction via the pericardium. Normally in series interaction, inspiration increases RVSV that is carried over with a two-three heartbeat delay to the LV [16,17]. The inspiratory increase in right heart venous return was simulated with a triangular, vena caval volume pulse as described in Experiment 1. The stiffened septum and fixed P_PERI _served to eliminate the immediate effects of V_RV _increase on the LV, allowing for quantification of the series effect on LVSV. With control effusion level, the volume pulse delivered to the right heart caused a 30.4% perturbational increase in RVSV. After 1.5 seconds, or roughly two heartbeats, LVSV increased by 3.3%. With 1000 ml effusion, RVSV increased by 87.8% due to the volume pulse, followed by 25.3% increase in LVSV 1.5 seconds later (see Table 5). Thus, through the series mechanism alone, there was a significant increase in LVSV variation during tamponade. However, the initial decrease of LVSV is not seen when parallel ventricular (septal) interaction is removed.

**Table 5:** Percent Changes in Stroke Volume in Isolated Ventricular Interactions

**V_PERI _(ml)**	**Series Ventricular Interaction**		**Parallel Ventricular Interaction**	
	
	*% Change in RVSV*	*% Change in LVSV*	*% Change in RVSV*	*% Change in LVSV*
15 (control)	30.4	3.3	41.1	2.6

1000 (severe tamponade)	87.8	25.3	40.0	28.9

Percent changes in RVSV and LVSV as a result of a triangular volume pulse delivered to the vena cava under breath-holding at mean P_PL_. The results for two experimental setups are shown, the first with isolated series ventricular interaction, the second with isolated parallel ventricular interaction for both control (15 ml) and severe tamponade (1000 ml) effusion levels (see text for details). The percent change in LVSV increases with tamponade. Furthermore, for the two effusion levels, the percent change in LVSV is of comparable magnitude for both types of interactions, demonstrating that both are equally significant interactions.

To isolate parallel ventricular interaction, control and severe tamponade simulations were run under conditions of constant pulmonary venous pressure with an independent constant pressure pump placed at the input to the LA. This virtual setup eliminates right heart serial influence on the left heart (i.e., venous filling conditions for the LV are constant). The triangular venous return pulse technique described earlier in Experiment 1 was again employed under conditions of normal septal stiffness and breath-holding at mean P_PL_. In order for a fair comparison of the LVSV percent variation in both isolated ventricular interaction setups, the chamber volumes in this experiment were scaled down to match the steady-state control stroke volume with that of the isolated series interaction control case. The percent change from steady-state in RVSV and LVSV was calculated for both the control and severe tamponade cases. With the perturbational venous volume increase under normal effusion level, RVSV increased by 41.1% and LVSV simultaneously decreased by 2.6%. With 1000 ml effusion, RVSV increased by 40.0% while LVSV decreased by 28.9% (see Table 5). The percent change in LVSV is greatly increased with tamponade. Comparing the isolated series interaction experiment with that of the isolated parallel interaction experiment, the percent variation caused by parallel interaction in severe tamponade is roughly the same as that for series interaction.

In severe tamponade, ventricular end-diastolic volumes tend to be 180° out of phase over the respiratory cycle [45]. Such a phasic relationship occurs as the result of parallel ventricular interaction and is due to the strong competition for filling space in tamponade. A sinusoidal breathing pattern was introduced for a clear calculation of the phasic relationship between ventricular end-diastolic volumes. The control case showed a phase difference between peak RVEDV and peak LVEDV of 138°. With heightened parallel interaction in severe tamponade, this phase difference increased to 167°. In a virtual experiment where P_LA _was controlled by a constant pressure source, thus eliminating the series effect, the phase difference increased further to 178°.

#### Section Summary

The involvement of ventricular interaction in pulsus paradoxus is examined by way of tracking the effect on both ventricles of a vena caval volume pulse equivalent to a single inspiratory venous return increase. Parallel and series type ventricular interactions are isolated to compare the contributions of each to respiratory variation in LVSV. Model results reveal equally significant contributions to variation in LVSV.

## Discussion

We have used our large-scale model of the H-CRS to simulate the compressive effects of cardiac tamponade on atrial and ventricular hemodynamics under conditions of normal and elevated tidal breathing. At levels of breathing consistent with observed breathing waveforms in severe tamponade [17], our modeling correctly simulates a wide range of hemodynamic waveform changes including chamber pressure equalization, partial RA chamber collapse and AV interaction (Figure 4), abnormal septal motion, abnormal and highly varying flows (Figure 7), and pulsus paradoxus (Figure 8). The ability to characterize the hemodynamic waveforms in some detail greatly facilitates biophysical interpretation and yields insight into mechanisms underlying cardiac tamponade. This is particularly true of the subtle components of the right atrial and pericardial pressure waveforms that change dramatically in tamponade.

It should be noted that our composite model represents a patient whose heart has normal characteristics based on various data sources but is encased in an abnormal pericardium of the patient described by Reddy et al. [3]. Therefore, qualitative and not quantitative correlation (i.e., Figure 3) is expected.

Our modeling study presents a unique view of tamponade ranging from the dynamics occurring in a single cardiac cycle and how they come to play over a cycle of respiration, including analysis of septal motion, valve flows, chamber volumes, and pressures. This is largely due to our characterization of the septum as a third contractile pump. This premise provides the platform for atrioventricular as well as right-left ventricular interactions. Our heart model thus yields accurate representations of ventricular pressures [11,12,37], including the specific septal contribution to RV systolic ejection, unlike the case when a passive septal model is used (see Figure 9). Moreover our work shows that with pericardial effusion, septal motion influences AV interaction, asynchrony of right and left heart ejection, and reduced ejection times, which are phenomena not demonstrated by the Sun et al. model [27]. In addition, the respiratory section of the model is used to provide predictions of gas exchange with pericardial effusion, yielding additional insights consistent with clinical observations on hypoperfusion. The deeper respiratory pattern used in this study with asymmetric emphasis on expiration aids in the oxygenation of peripheral tissue, reducing the hypoxic and hypercapnic effects of severe tamponade. These elementary observations point out the need for in-depth observation of spontaneous breathing patterns in tamponade patients as well as laboratory experiments that examine ventilation patterns and effectiveness of gas transport in tamponade.

### Atrioventricular Interaction

In the thin-walled right heart, clinically observed features such as an elevated P_RA _and lack of the y-descent feature [6] have been shown to be sensitive indicators of the effect of pericardial constraint and increased AV interaction [19]. The display of model-generated hemodynamic variables shown in Figure 4 and Figure 5 allow us to interpret AV interaction and its enhancement by septal motion and the production of pulsus paradoxus at normal levels of breathing. With pericardial constraint, the pattern of atrial filling is largely dependent on ventricular size, and hence the septal position. Systolic ventricular emptying allows for maximum atrial filling. The systolic interval has two parts: PEP and ejection. In tamponade, LPEP increases due to reduced preload [38] whereas RPEP is relatively unaffected. Since the start of systole is the same for both ventricles, this produces a disparity in the start of ventricular ejection times, slowing aortic valve opening and delaying septal motion. This produces a largely labored systolic atrial filling, with maximum atrial volume when the septum is maximally leftward. Hence, the pericardial constraint that produces the lock-step atrial filling with the systolic dip of the P_PERI _waveform and ventricular filling with the diastolic dip is further impacted by septal motion. Elevated P_PERI _steers chamber pressures to undergo similar variations, particularly in the relatively more compliant right heart. In systole, the pericardial systolic dip draws P_RV _down as well, causing a premature pulmonic valve closure and early tricuspid flow. Normally, septal rightward motion upon deactivation administers a final aid to RV stroke output; however, due to the premature cessation of RV systole and commencement of tricuspid flow, this septal rightward thrust does no more than briefly interrupt RV filling. Therefore, pericardial constraint introduces asynchrony of outlet and AV valves, altering the nature and stroke volume impact of septal motion, respectively. Our simulation studies also show that pericardial constraint alone produces abnormality in septal mechanics regardless of the level of respiration (Figure 13), but to even greater degrees with deeper levels of breathing.

### Flow Abnormalities

Changes in flow due to tamponade were demonstrated with the H-CRS model. Flow respiratory variation becomes equally significant in both sides of the heart (Figure 7). Venous flows exhibit lower D/S ratios with increased AV interaction. In transvalvular flows, there is a clinically observed reduction [1,34] in the E-wave magnitude due to impaired filling in early diastole and there is a more dominant A-wave in late diastole. In severe tamponade (1000 ml), our simulations predict the existence of a split E-wave. We have explained the genesis of the early component of the split E wave in connection with Figure 11, implicating early relaxation of the RV pressure waveform and septal movement. The split E-wave occurs only in the tricuspid flow waveform, only at high levels of effusion (1000 ml and greater), and at low P_PL_. To our knowledge, the split E-wave phenomenon has not been documented in the clinical literature, and therefore it remains a model prediction that should be examined more closely in future studies.

### Signs of Pulsus Paradoxus

With tamponade, respiratory-associated hemodynamic fluctuation is exaggerated in the left heart, and is increasingly out of phase with the right heart, signaling pulsus paradoxus. It has been noted that deep tidal levels of inspiration exaggerate pulsus paradoxus [8,18,31,34,46]. Elevated venous pressures in tamponade, combined with lowered pleural pressures during inspiration, causes a relatively larger atrioventricular pressure gradient in early diastole, and hence greater fluctuations in ventricular filling with respiration compared with control. Our simulations confirm this with exaggerated flow and pulse pressure variations.

Pulsus paradoxus is also a common occurrence in asthma patients, who tend to exhibit wide respiratory excursion and low pleural pressures on inspiration [31]. Respiratory pattern changes are often associated with tamponade, i.e., dyspnea (shortness of breath) [6]. Deeper spontaneous respiration has been observed in dog experiments with induced pericardial effusion [17,34]. The influence of respiratory pattern on the level of pulsus paradoxus leads to possible future model investigation of the respiratory-related onset of pulsus paradoxus in cases such as asthma.

### Mechanisms of Pulsus Paradoxus

Historically, pulsus paradoxus has been explained in several ways including: (a) diaphragmatic descent on inspiration causing an increase in pericardial pressure which impedes LV filling [47]; (b) failure to transmit pleural pressure changes to the left heart by a taut pericardium that results in lowered pulmonary venous return [46,48,49]; (c) increased ventricular interdependence due to competition for filling space which increases respiratory variation [45]; (d) increased trans-pericardial pressure on inspiration which lowers LV filling [17,45]; and (e) inspiratory leftward septal bulge which decreases LV ejection [18].

It is generally agreed that in tamponade with a normal breathing pattern, inspiration produces an increase in right heart venous return and subsequently the left heart phenomenon of LVSV reduction [7,10,17]. This statement describes the phenomenon of pulsus paradoxus, but not the underlying mechanisms. Our modeling studies dig deeper to explore putative mechanisms and we propose that pulsus paradoxus in tamponade is the result of two types of exaggerated ventricular interaction (series and parallel), as well as AV interaction for each heart that changes the operating characteristics of the ventricles. In particular, series interaction plays a dominant role in producing pulsus paradoxus as the normal respiratory variation in filling volumes becomes significant with overall lowered stroke volume in tamponade. Parallel interaction is a result of space limitations imposed by pericardial constraint, increasing ventricular interaction both via the septum and via the pericardium. The manifestation of this dominant pericardial constraint as it affects chamber mechanics is AV interaction. Pericardial constraint increases AV interaction and results in lowered filling, decreasing LV preload and delaying leftward septal movement on systole; this shortens LVET and further reduces stroke volume, while the abnormal septal motion enhances AV interaction. RVET is also shortened with premature RV relaxation causing early pulmonic valve closure, and a reduction in RVSV. Thus, AV interaction in tamponade affects chamber mechanics during diastole directly, which carries through to affect ejection dynamics and the septal pathway by which the ventricles interact. The second aspect of parallel interaction is ventricular interaction via the pericardium. Localized, respiratory filling variation in one ventricle is transmitted to the pericardium as a pressure change, and hence transmitted to the other ventricle via the pericardium. Respiration-induced venous filling also has an important additional effect on septal position and consequently the swing of septal pumping. In tamponade, the leftward thrust during systole is delayed further on inspiration, bringing LVSV to a minimum, whereas RVSV is maximum at this point with greatest end-diastolic volume and RVET. The parallel ventricular interaction mechanism alone causes a near 180° phase difference between the RV and LV volumes in tamponade.

Experiment 2 (Results section) on *Isolated Series and Parallel Ventricular Interactions *shows that these two mechanisms for ventricular interaction cause a significant respiratory variation in LVSV with tamponade. By virtue of the matched stroke volumes in control cases for the isolated series and isolated parallel interaction experimental setups, the contribution of each mechanism to LVSV variation could be evaluated. This comparison shows that the contribution by the parallel mechanism (28.9% variation) is roughly the same as that of the series mechanism (25.3% variation) (Table 5).

### Theories Regarding Other Mechanisms

Other mechanisms for production of pulsus paradoxus are quoted in various studies. Golinko, et al. [48] and Ruskin, et al. [46] support the theory that LV filling is impeded by a lowered and/or reversed pulmonary venous to LA pressure gradient on inspiration, which is accompanied by pulmonary blood pooling. Our simulations predict that pulmonary blood pooling is due to constriction of the cardiac chambers. As seen in Figure 5, our virtual experiments show that pulmonary venous reversal flow actually decreases with effusion. Furthermore, an experiment not detailed in this paper shows that the phasic relationship between RVEDV and LVEDV changes for various breathing frequencies indicating LV volume fluctuation is independent of pleural pressure, and dependent rather on RV volume fluctuation. Shabetai, et al. [17] demonstrates that LVSV decrease in pulsus paradoxus is a right-sided phenomenon that is manifested in the left heart. Our model results show right-sided contributions to the left heart respiratory variation via the pulmonary vascular pathway, via the pericardium, and via the septum. However, left heart AV interaction is also observed in our model, in which pericardial constraint independently alters LA and LV mechanics, thus contributing to the nature of the pericardial pressure waveform and hence septal movement.

Another theory is that inspiration during tamponade creates an increased pressure afterload [8,48,50]. Simulations not included in this paper have examined the pressure difference between ventricular pressure and arterial pressure during systolic ejection, to look for an increase in this pressure difference ΔP as an indicator of increased afterload. Although an observable variation with respiration is seen in the left heart, with maximum ΔP at inspiration, the overall ΔP level is reduced when compared to the control case. The model predicts that this systolic effect of afterload is not increased with tamponade. Our studies show that in tamponade the fundamental mechanism in LVSV reduction is reduction in LV preload from pericardial constraint. It is further reduced by inspiration due to ventricular interaction, which in turn lowers LVSV. The underlying mechanism involved in systole is a variation of LPEP that is fundamentally attributed to AV interaction from pericardial constraint and modulated by respiration. One might observe this reduction in LVSV in inspiration and attribute it to an increased afterload presented to the ejecting ventricle. However, our simulations suggest that the mechanism for reduced stroke volume is the change in the operating characteristics of the septum and the influence that respiration has on those same mechanical characteristics. The septum after all is a vital part of the important parallel ventricular interaction pathway.

## Conclusion

Large-scale integrated models of the human cardiovascular and respiratory systems can provide a means of analyzing complicated problems associated with critical care medicine. Cardiac tamponade with pulsus paradoxus is only one example. Our H-CRS model has allowed us to dissect the problem into two aspects: 1) pericardial constraint modifying ventricular and septal mechanics, hence, lowering stroke volume, and 2) ventricular interaction relating to how respiration varies RV and LV stroke volume.

Our model offers two explanations for lowered stroke volume seen in cardiac tamponade. They are independent effects of pericardial constraint, one occurring in diastole and the other occurring in systole. In diastole, pericardial constraint causes overall filling volume constraint, due to both ventricular constriction and AV interaction, which reduces the overall volume available from the atria. The reduction of filling volume contributes to a reduction in stroke volume. In systole, pericardial constraint causes reduced LV preload, resulting in abnormal septal movement in early systole and premature reduction in P_RV_. These effects prolong LPEP, reduce RV and LV ejection times, and thereby reduce stroke volume. Thus, with the aid of the model, we have been able to break down pericardial constraint into diastolic and systolic components. The diastolic effects are well known in literature, but our simulations suggest that the systolic effects come about because of AV interaction determining LV preload.

Respiratory variation in stroke volume manifests itself as pulsus paradoxus, and we have identified parallel and series ventricular interactions as mechanistic explanations. Specifically, parallel interaction occurs with respiratory fluctuation of right heart inflow because of filling volume constraint (imposed by the septum and pericardium) and abnormal septal movement. These are both a direct result of AV interaction stemming from pericardial constraint. Likewise, respiration controls septal movement, favoring one heart over the other in systolic ejection. Series interaction exists due to the nature of the series blood pathway, but the magnitude of filling volume variation is exaggerated in tamponade due to lower stroke volumes. The end result of these multifaceted parallel and series interactions is a 180° phasic relationship between the ventricular end diastolic volumes. Importantly, we show both these interactions have an equal and significant contribution to LVSV respiratory variation, and hence to pulsus paradoxus.

Our H-CRS model has the following impacts to the field of tamponade study:

(a) It fully describes the clinical hemodynamic spectrum of tamponade including right heart signs, pulsus paradoxus, transvalvular flow variation at the cardiac inlets and outlets, and cardiac output compromise;

(b) It introduces the concept that fluid pericardial pressure in tamponade directly modulates cardiac chamber dynamics in both diastole and systole;

(c) It quantitatively dissects the exaggerated right-left ventricular interaction, atrioventricular interaction, and the pleural (respiratory) pressure-mediated hemodynamic changes that is characteristic of pulsus paradoxus in tamponade;

(d) It introduces the concept that septal motion and septal characteristics directly affect ventricular interaction (i.e., septal motion is responsible for ventricular desynchronization in tamponade;

(e) It produces a full hemodynamic and respiratory analysis of tamponade which may serve as a roadmap for future study of pericardial diseases.

## Abbreviations

H-CRS: human cardiovascular-respiratory system; P-V: pressure-volume; RA: right atrium; LA: left atrium; RV: right ventricle; LV: left ventricle; SPT: septum; PERI: pericardium; TCV: tricuspid valve; PAV: pulmonic (arterial) valve; MV: mitral valve; AOV: aortic valve; PO: pulmonic valve opening; PC: pulmonic valve closure; AO: aortic valve opening; AC: aortic valve closure; S: systole; D: diastole; A_R_: reversal wave; AV: atrioventricular; PEP: pre-ejection period; SV: stroke volume; CO: cardiac output; HR: heart rate; MAP: mean arterial pressure; EDV: end-diastolic volume; RPEP: right ventricular pre-ejection period; LPEP: left ventricular pre-ejection period; RVSV: right ventricular stroke volume; LVSV: left ventricular stroke volume; RVET: right ventricular ejection time; LVET: left ventricular ejection time; λ: pericardial stiffness parameter; P_RA_: right atrial pressure; P_LA_: left atrial pressure; P_RV_: right ventricular pressure; P_LV_: left ventricular pressure; P_PERI_: pericardial pressure; P_TPERI_: transmural pericardial pressure; P_PL_: pleural pressure; P_PA_: pulmonary arterial pressure; P_AO_: aortic pressure; P_0_: pericardial pressure parameter; V_RA_: right atrial volume; V_LA_: left atrial volume; V_RV_: right ventricular volume; V_LV_: left ventricular volume; V_PERI_: pericardial volume; V_H_: total heart volume; V_0_: pericardial volume offset; V_SPT_: septal volume; Q_VC_: vena cava flow; Q_PV_: pulmonary venous flow; Q_TC_: tricuspid flow; Q_M_: mitral flow; Q_PA_: pulmonary arterial flow; Q_AO_: aortic flow; PO_2_: O_2 _partial pressure; PCO_2_: CO_2 _partial pressure; SO_2_: Percent O_2 _saturation; A-V: arterio-venous.

## Competing interests

The authors declare that they have no competing interests.

## Authors' contributions

DR carried out the tamponade modeling studies and drafted the manuscript. CL significantly contributed to the development of the human cardiopulmonary model and was involved in the analysis of data. TSM made substantial intellectual contributions to the study and in drafting of the manuscript. JWC made key contributions to the conception and design, analysis and interpretation of data, and drafting of the manuscript. All authors read and approved the final manuscript.
